# Generative vs. Discriminative Recognition Models for Off-Line Arabic Handwriting

**DOI:** 10.3390/s18092786

**Published:** 2018-08-24

**Authors:** Moftah Elzobi, Ayoub Al-Hamadi

**Affiliations:** Institute for Information Technology and Communications (IIKT), Otto von Guericke University, 39106 Magdeburg, Germany; ayoub.al-hamadi@ovgu.de

**Keywords:** Arabic OCR, offline handwriting recognition, HMM, CRF, HCRF

## Abstract

The majority of handwritten word recognition strategies are constructed on learning-based generative frameworks from letter or word training samples. Theoretically, constructing recognition models through discriminative learning should be the more effective alternative. The primary goal of this research is to compare the performances of discriminative and generative recognition strategies, which are described by generatively-trained hidden Markov modeling (HMM), discriminatively-trained conditional random fields (CRF) and discriminatively-trained hidden-state CRF (HCRF). With learning samples obtained from two dissimilar databases, we initially trained and applied an HMM classification scheme. To enable HMM classifiers to effectively reject incorrect and out-of-vocabulary segmentation, we enhance the models with adaptive threshold schemes. Aside from proposing such schemes for HMM classifiers, this research introduces CRF and HCRF classifiers in the recognition of offline Arabic handwritten words. Furthermore, the efficiencies of all three strategies are fully assessed using two dissimilar databases. Recognition outcomes for both words and letters are presented, with the pros and cons of each strategy emphasized.

## 1. Introduction

Even though deep-learning neural network (DNN) approaches perform excellently in various machine-learning domains such as computer vision, statistically-based strategies continue to draw interest. This is particularly the case in the field of optical character recognition. Initial training of high-performing DNNs typically requires large amounts of grounded data, in contrast to statistical strategies. The collection and annotation of enough examples of offline handwriting for DNN training is both labor-intensive and slow. Furthermore, training is very costly for tasks that require appreciable resources such as powerful GPUs and comparatively long periods of time (weeks or more) [1]. The situation compelled us to resolve the problem regarding offline Arabic handwritten word recognition in terms of a probabilistic sequence labeling approach. This research therefore aims to assess the performances of probabilistic classification strategies that are useful for labeling sequences of feature sets extracted from offline Arabic handwriting. First of all, we presented a generatively-trained Hidden Markov Model (HMM) method constructed directly over explicit Arabic handwriting segmentation components, as described in [2]. So as to lower the numbers of class labels, a primitive features-based taxonomization method as initially recommended in [3] was further implemented. The strategy uses elements including segment numbers and the presence of loops in images for taxonomizing letter forms accordingly, thus reducing label numbers from 104 down to a maximum of 42 within four different categories. The suggested paradigm employs dual sequential feature sets to define the shapes of segmented units along two differing directions, i.e., anticlockwise and clockwise. Unlike prior HMM methods that accord with offline handwritten word recognition strategies, the key innovations of our HMM recognition strategy are as follows: (i) instead of depending on implicit segmentations as by-products of HMM decoding processes, we incorporated explicit segmentation phases into our recognition strategies for segmenting words into their letter representatives; (ii) rather than utilizing conventional sliding, windows-based feature sets subsequent to properly-sized normalization and thinning, we extracted dual sequential, shape-representative feature sets from segmented images; (iii) for detection, we implemented left-to-right banded topologies in the construction of HMM classifiers and further equipped these with adaptive threshold models that derive from the merging of every trained model comprised of all letters. The primary function of adaptive thresholds is to enhance efficiencies by enabling reasonable rejections of meaningless areas-of-pixel that can occur noisily in either or both image-acquisition and segmentation phases. Second, we presented alternatives to generative paradigms in the form of dual probabilistic discriminative-based classifiers, i.e., linear-chain CRF and HCRF extensions. These methods have been suggested and were successfully implemented recently in numerous sequence labeling problems, such as computer vision, natural-language processing and bioinformatics [4]. This is due to the fact that either strategy assumes no dependencies among inputs and allows representations of the complex relationships among observations and to-be-predicted labels. As with our HMM-based strategy, the recommended CRF and HCRF classifiers incorporate the same explicit segmentation component and execute taxonomization procedures before detection.

## 2. Previous Works

This section presents a survey of related research, with a focus on strategies that embrace discriminative and generative paradigms via probabilistic modeling. Based on the literature, generatively-trained HMM-based strategies are generally in widespread usage in the area of offline handwritten word recognitions [5,6,7]. The first section will therefore briefly present suggested HMM-based strategies. By incorporating right-to-left discrete HMM, as well as Kohonen self-organizing maps for feature quantization, Dehghan et al. [8] pioneered demonstrations of the viability of applying HMM methods for holistic identification of offline Farsi and Arabic handwritten words. Experiments were carried out using a dataset that was comprised of the names of cities in Iran, while sliding window-derived feature vectors were arranged from histograms of chain-coded contour directions. Observed accuracy rates using the method reached 65%. Pechwitz and Maergner [9] employed the 1D semi-continuous-HMM-based strategy for the detection of handwriting, wherein sliding window-derived feature vectors were obtained from normalized grey images of words. Before feature sets were passed to HMM, Loeve-Karhunen transforms were applied to decrease dimensionality. In addition, IFN/ENIT information was utilized for testing and training, and maximum recognition rates of 89% were observed. For the detection of offline Arabic handwriting, Al-Hajj et al. [10] suggested an arrangement of three right-to-left HMM classifiers. All classifiers were built upon specific sliding window orientations in order to surmount the significant difficulties of offline handwritten words, including overlaps, inclinations and the shifted positions of diacritics. Testing and training was carried out using IFN/ENIT information, with recognition decisions generally attained via experiments with combinations of various methods, such as majority vote and sum rules combined with neural network learning, on all three classifier results. The highest recognition rates attained reached 90%, wherever neural-network combinations were chosen. Dreuw et al. [11] suggested discriminatively-trained HMM for the detection of Arabic and Latin offline handwriting. In place of common expectation maximization (EM) training methods, the researchers proposed minimum phone error (MPE) and maximum mutual information (MMI) methods for training holistic modeling paradigms. The Arabic IFN/ENIT and the English IAMdatabases were deployed for assessing the approach, and the error-rates are lowered by 33% and 25%, respectively, in comparison to the EM. To recognize Arabic handwriting obtained from IFN/ENIT databases, Ahmad and Fink [12] suggested a dual-stage, HMM-based strategy. Given the advantage that numerous Arabic letters share the same main bodies and are differentiated only through diacritics, it was decided that diacritics and primary strokes would be modeled discretely. Accordingly, the variety of schemes was considerably decreased, and the researchers were able to achieve results comparable to those obtained in related studies. Seeing the successful implementation of these methods in numerous fields, i.e., bioinformatics, natural-language processing and computer vision, many researchers recently proposed CRF for use in the identification of offline Chinese and Latin handwriting. As far as we know, our prior research [13] remains the first published study that examined the utility of CRF and HCRF in the identification of offline Arabic handwriting. This research therefore extends the original research paper that was conveyed during the conference. Due to the absence of published research on the proposed CRF and HCRF for the identification of offline Arabic handwritten words, we briefly summarize similar strategies for Chinese and also Latin manuscripts. In earlier related research, Feng et al. [14] examined and compared the efficiencies of CRF and HMM methods in word recognition tasks that focused on handwritten historical manuscripts. A discrete feature set was obtained from 20 pages of documents written by George Washington and utilized for training and evaluating CRF-based and also HMM-based classifiers. The researchers carried out many experiments with various beam search techniques so as to hasten training courses of CRF classifiers, and the findings showed that the CRF method outperforms HMM. Nonetheless, it was discovered that to boost efficiency, it was necessary to decrease state spaces through the application of the CRF method at the character level, which was implemented in our recommended approach. Hamdani et al. [15] examined the performances of segmental CRF (SCRF) in tasks involving large-vocabulary English handwritten word recognition, wherein multi-layer perceptron networks and LSTM-RNNs were utilized to produce observations. The efficiency of the recommended method attained a 13.7% reduction of writing errors in comparison to a baseline model built with the RWTH-HWRmodule. In an integrated scheme for the detection of Latin handwriting, Chen et al. [16] suggested that Boltzmann machine-trained deep neural networks be combined with linear CRF. With this strategy, deep networks were utilized to produce non-linear latent feature sets, which were then passed on to the CRF for recognition. The recommended method was tested on dual handwriting datasets and reportedly outperformed methods that adopt shallow CRF. Zhou et al. [17] proposed a method for detecting Japanese and Chinese text that accorded with semi-Markov CRF. The researchers began with descriptions of semi-CRF on lattices comprised of every possible segmentation-recognition hypothesis of strings, in order to directly approximate the a posteriori probabilities for each. CRF features were functionally defined over linguistic and geometric information from character recognition, with the negative log-likelihood utilized to optimize modeling of the parameters. At the character level, the corresponding recognition rates attained for Chinese and Japanese handwriting were 95.20% and 95.44%.

## 3. Shape Descriptions Features for Arabic Handwriting Recognition

Features might be chosen to represent either the stroke external characteristics, i.e., the boundary, or to represent the internal characteristics that are the pixels contained within the stroke’s image. External representation is usually more appropriate when focusing on the shape characteristics, whereas internal representation is the better choice when the focus is on characteristics relevant to color or texture [18]. Related literature contains many different types of features that might be geometric (e.g., geometric moments, directional histograms, etc.), structural features (e.g., topological features, Fourier’s descriptors, etc.) or space transformation features (e.g., principal component analysis, linear discriminant analysis, etc.) [19]. Due to the fact that HMM, CRF and HCRF are especially powerful for the task of sequential feature classification, features extracted from a letter or a word image should be sequential or can be easily converted into a sequence that eventually is passed to the appropriate classifier for recognition. The most widely-adopted sequential features for offline handwriting recognition are those extracted using the principle of the so-called sliding window [6,10,14,17]. Typically, these types of features are sequences of observations extracted by shifting a window along the image of the word from right to left or vice versa.

In the case of Arabic handwriting, the sliding window is shifted a small distance from the right to the left, and for each position. a feature vector is extracted [20]. When using sliding window-based features, typically, a large number of features are primarily extracted. Then, to get rid of redundant and irrelevant features, feature selection or reduction algorithms are usually needed to allow the recognition process to be computationally tractable. Furthermore, sliding window-based features are particularly adequate to represent the image local patches (local details), rather than capturing shape global characteristics (e.g., contour curvature), which prove to be very important for handwriting recognition.

Additionally, being computed based on the pixel’s connectivity to local image neighborhoods, sliding window features are sensitive to the stroke width and relatively more prone to multiple handwriting distortions (e.g., slant, skew, etc.). Inspired by the works of [21,22,23], we propose a robust, yet simple approach for extracting two sets of shape descriptor features, which are proper to be used as an input for a sequence labeling-based recognition system. Besides avoiding the above-mentioned drawbacks of sliding window features, the proposed features have a number of desirable characteristics such as:

- Less expensive to extract and to process.
- Capturing the letter’s distinctive shape characteristics.
- Invariant to stroke width and less sensitive to handwriting distortions.
- Easily converted to vectors of observations suitable for sequence classifiers.

### 3.1. Extraction of Feature Descriptors

Since our main objective is to build an unconstrained recognition system, our feature extraction module is built on top of an explicit segmentation method detailed in [2]. Provided that writing styles differ greatly with respect to height, width, skew and slant, feature extraction begins by normalizing the handwritten word against handwriting deformations, i.e., skew and slant. Then, to further minimize the within-class variations, segmented images are size-normalized while preserving the aspect ratio.

**Figure 1:**
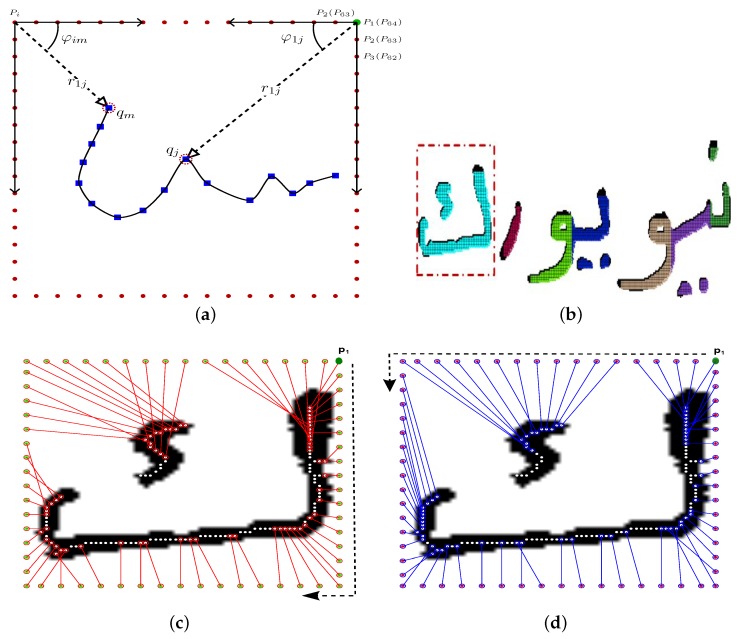
Extraction of features descriptors: (**a**) extracted features computation; (**b**) segmented handwritten word; (**c**) clockwise features; and (**d**) anticlockwise features.

For size normalization, a backward linear normalization method is employed to map the pixel coordinates of all segment images (usually of different sizes) into a standard plane of fixed N×N dimension where N=64 is found to be optimal [24]. Figure 1 depicts the core idea of our approach, where feature extraction starts by uniformly distributing a set $P=\{p_{1},p_{2},\ldots ,p_{m}\}$, $p_{i}\in R^{2}$, of *m* reference points along a rectangle that tightly contains the thinned image (see Figure 1a). Typically, *m* can be any natural number less than or equal to *n*, where *n* is the total number of pixels constituting the thinned image. Moreover, in practice, *m* should be proportional to the size of the normalized images; therefore, m=64 is chosen. Since the main focus of this work is on Arabic handwriting, the first reference point $p_{1}$ is positioned on the rectangle’s upper-right corner.

Additionally, Let $Q=\{q_{1},q_{2},\ldots ,q_{n}\}$, $q_{i}\in R^{2}$ be the set of pixels coordinates of the thinned image. Starting at the reference point $p_{1}$ and in both clockwise and anticlockwise directions, for every $p_{j}\in P$, we search for the nearest $q_{j}\in Q$. Upon identifying $q_{j}$ and by using $p_{i}$ as the pole, we estimate the radial distance $r_{ij}$ and the angle $\varphi_{ij}$ according to Equation (1), and eventually, we exclude the pixel coordinate $q_{j}$ from the pixel set Q.

(1) $$r_{ij}=\sqrt{{\left({\overset{´}{x}}_{j}-x_{i}\right)}^{2}+{\left({\overset{´}{y}}_{j}-y_{i}\right)}^{2}},\varphi_{ij}=\tan^{-1}\left(\frac{{\overset{´}{y}}_{j}-y_{i}}{{\overset{´}{x}}_{j}-x_{i}}\right),\mathrm{where}\;\;p_{i}=\left(x_{i},y_{i}\right),\;q_{j}=\left({\overset{´}{x}}_{j},{\overset{´}{y}}_{j}\right)$$

Algorithm 1 outlines the feature extraction process, where each $p_{i}\in P$ is assigned one and only one $q_{j}\in Q$, and as a result, two different feature descriptor vectors $\chi_{a}=\left(\chi_{1},\chi_{2},\ldots ,\chi_{64}\right)$, $\chi_{c}=\left(\chi_{1},\chi_{2},\ldots ,\chi_{64}\right)$, where $\chi_{i}=\left(r_{i},\varphi_{i}\right)$, are constructed from every $\left(p_{i},q_{j}\right)$ pair in anticlockwise, as well as in clockwise directions, respectively. In this context, it is also important to state that computation is performed with the assumption that n≥m, i.e., the number of pixels *n* in the segment skeleton image is greater than or equal to the number of reference points *m*.
**Algorithm 1:** Extraction of feature descriptors.
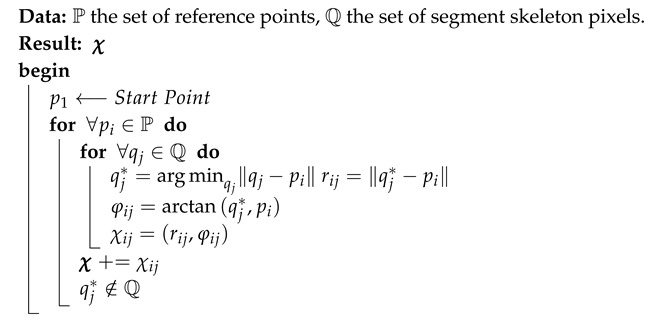


**Figure 2:**
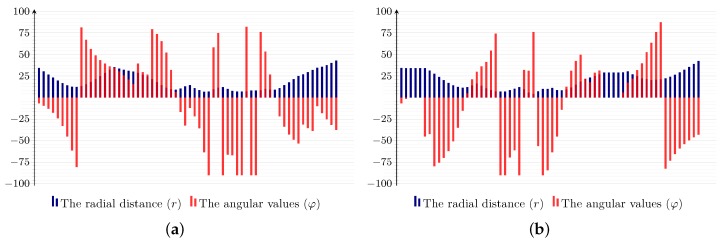
Shape descriptor profiles for letter ك: (**a**) the profile in the clockwise direction; (**b**) the profile in the anticlockwise direction.

Figure 1 illustrates further the proposed feature extraction step, where Figure 1a shows how features are calculated using reference points, as well as image pixels. Figure 1b presents a segmented handwritten word image using the method presented in [2]. To improve the robustness of the recognition system against various writing distortions, we computed the features in two different directions; Figure 1c,d, depicts the results along the two directions for letter ك “KAF” (enclosed in the red rectangle) in Figure 1b. To demonstrate the feature descriptors discriminatively, Figure 2 shows the feature profiles for letter “ك” in the two mentioned directions.

### 3.2. Vector Quantization for Feature Sequences

Basically, quantization is the process of mapping a large number of different input values into a far smaller set of discrete values [25]. In the case of vectors of data points, most quantization algorithms work by clustering nearby vectors into groups (clusters). A widely-used quantization technique is the k-means clustering algorithm, thanks to its relatively fast convergence property [26]. Its drawback, however, is the sensitivity to the initialization step, meaning that the algorithm’s performance is fully determined by the initial values. Thus, a reasonable solution can only be achieved through initialization values that lie close to a good clustering solution. To overcome this problem, we adopted a k-means initialization method proposed in [25]. In this method, the authors suggested to use the affinity propagation algorithm (AP) to initialize the k-means clustering. AP works initially by assuming that all data points are potential exemplars that iteratively exchange messages until a satisfactory clustering solution is reached. Without any assumptions about the feature data distributions and in order to estimate the number of clusters for a given letter, the AP algorithm starts by constructing a similarity matrix. Where elements of the matrix are pairwise similarity values between each data point $s(\chi_{i},\chi_{k}),\chi \in \chi$, typically similarity values quantify how well $\chi_{k}$ is suited to be the exemplar of $\chi_{i}$.

Like in [25], we simply define the similarity function *s* as the negative squared Euclidean distance between data points. Then, for each letter, the AP is applied twice, once for $\chi_{a}$ and once for $\chi_{c}$, and the average of the estimated number of clusters is computed. This process is performed for every letter in every form, and the number of clusters (i.e., the number of quantization levels) or *k* of k-means is calculated as the overall average of all clusters of all letters, where k=16 is found to be the optimal number of quantization levels. Eventually, a k-means clustering algorithm with k=16 is used to quantize the feature descriptors $\chi_{a}$ and $\chi_{c}$. As a result, we obtained two vectors of observation sequences $f_{a}$ and $f_{c}$ of length 64, containing values of observed quantization level indices, where $f_{a}=\left(f_{a_{1}},f_{a_{2}},\ldots ,f_{a_{64}}\right)$, with $f_{ai}\in \left\{1,2,\ldots ,16\right\}$ representing the anticlockwise observed feature sequence and $f_{c}=\left(f_{c_{1}},f_{c_{2}},\ldots ,f_{c_{64}}\right)$, with $f_{ci}\in \left\{1,2,\ldots ,16\right\}$ representing the clockwise observed feature sequence.

## 4. Shape-Based Letters’ Taxonomization

**Figure 3:**
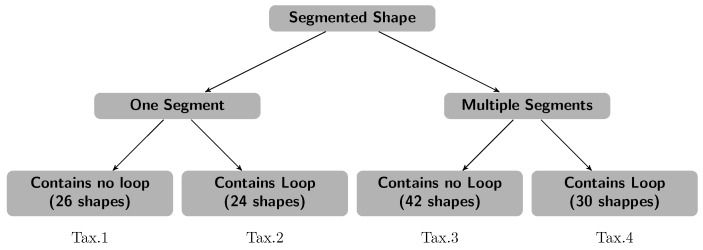
Letters’ taxonomy, according to the number of segments and the existent of loop(s).

The recognition of handwritten Arabic words is, firstly, reduced (through the explicit segmentation) into the problem of recognizing a segment that may represent a letter in a word. However, the Arabic alphabet contains basically 30 letters, and a letter may appear in two to four distinct shapes according to its position in a word, resulting in 104 substantially different shapes. A straightforward approach to alleviate this problem is to taxonomize letter shapes according to very primitive properties. Figure 3 illustrates the adopted taxonomy, which classifies letters according to the number of segment and whether they contain a loop(s) or not. Thereby, for example, letters such as د, ر, ﺍ, س, ح will be grouped under the first group from the left in Figure 3 (i.e., one segment and no loop group Tax.1), letters such as و, ﻣ, ح, ﻫ under the second group (i.e., Tax.2) and letters such as ب, ت, ث, ك in the third group (Tax.3), and the fourth group (Tax.4) will contain letters such as ﺽ, ق, ة, غ.

By following such a simple pre-classification step, we reduce the space of labels from 122 to 42 at most. Furthermore, notice that the total number of labels is 122 (not 104), because some letters appear twice in two different taxonomies, since they may be either written with or without a loop (e.g., م and ﻣ) or in one or two strokes (e.g., ﻜ and ك). depending on the personal writing style.

## 5. HMM-Based Recognition of Arabic Handwriting

This section describes in detail the proposed HMM-based recognition approach. Firstly, we show how the letter models are constructed and how the HMM parameters are initialized. Then, we explain the process of building the threshold model, and eventually, we present how recognition is performed. Basically, the system is built up of two sub-classifiers corresponding to the different types of features. Each sub-classifier contains an HMM model for each letter in each form, thereby each sub-classifier consists of 122 different HMM models. To cope with the errors of segmentation that negatively affect the system performance, a big model for each sub-classifier is constructed by ergodically connecting all other models. This model is called the threshold model and is dedicated to reject out-of-vocabulary segments.

### 5.1. Topology and Hidden States’ Optimization

**Figure 4:**
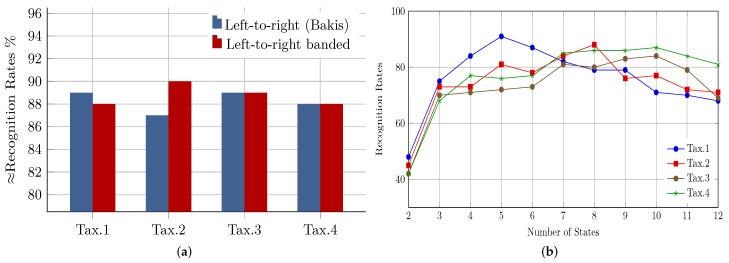
HMM topology and model size optimization: (**a**) left-to-right (Bakis) topology vs. left-to-right banded topology performance; (**b**) effect of HMM model size on performance.

The first step in building an HMM model is to specify the model topology and the number of model states and to keep them unchanged throughout the training phase. There are multiple different types of HMM topologies; however, there is no theoretical framework that can be used to determine the optimum topology. Nevertheless, when topologies produce similar results, then the simple one is the best, since it involves the fewest number of parameters that need to be optimized. According to [27], the most commonly-used topologies in the field of optical character recognition in general are the banded left-to-right and the left-to-right (Bakis) topologies. To assess and compare the performance of the two topologies, while the number of states is assumed to be globally fixed (i.e., eight states), dedicated HMM models are built for three randomly-chosen letters from each leaf node within the taxonomy. Since there are no boosts in performance justifying the use of Bakis’ topology (see Figure 4a), consequently, the simpler left-to-right banded topology is adopted.

In addition to choosing the proper topology, the number of states (the model’s size) for each HMM model should be carefully determined. Typically, there are two different paradigms that might be followed to select the number of states for the HMM model [28]. According to the first, the number of states should be proportional to the number of strokes within a handwritten word or a letter; whereas, in the second, the number of the model states is estimated as the average of the length of the observation sequence of the corresponding word or letter.

Due to the fact that we are converting feature data into equal-length vectors of observation sequences, estimating the number of states based on the length of the observation sequence will be equal to assigning the same number of states to all models, which can be an excessive or insufficient number for the respective letter model. Furthermore, for robust and efficient modeling, typically, letters consisting of multiple segments and complex shape characteristics require models with a bigger number of states, compared to one segment and simply-shaped letters. Hence, and in contrast to most related works, in which a global fixed number of states is optimized and used to model all letters, we optimized the model size for each node within the taxonomy separately. To estimate the optimal model size for a leaf node of letters (e.g., multiple segments with a loop), for every letter within the node, eleven different HMM models are created and trained, starting with a two-state model up to a twelve-state model. Moreover, a smaller portion of data (i.e., 30%) is used for testing, and the average recognition rates for all letters for every number of states are calculated. Ultimately, the number of states corresponding to the maximum recognition rate (the average) is selected as the optimal model size for all letters belonging to the considered node. Accordingly, Figure 4b shows the achieved recognition rates with respect to the different numbers of states in each node. Letters under Tax.1 (i.e., one segment and no loop), for example, reach the optimal performance with models of a size of five states, and this is justifiable given their simple shape characteristics; whereas the relatively complex shape characteristics of Tax.3 (multiple segments and no loop) and Tax.4 required models of 10 states in size to achieve the best results.

### 5.2. HMM Parameters Initialization

In addition to optimizing the model topology and the model size, several related works [3,29,30] confirm the fact that proper initialization of HMM parameters (i.e., the matrix of state transitions probabilities *A*, the matrix of observations probabilities *B*, and the vector of initial state probability distribution π) is positively affecting the overall system performance. Given that we adopted the banded left-to-right topology, matrix *A* is initialized according to Equation (2), which shows the corresponding mathematical general structure. In *A*, the diagonal probabilities represent the self-transitions and, those directly above the diagonal are used for the transition probabilities to the subsequent states.

(2) $$A=\left(\begin{array}{ccccc} a_{11} & 1-a_{11} & 0 & \cdots & 0\\ 0 & a_{22} & 1-a_{22} & \cdots & 0\\ \vdots & \vdots & \ddots & \vdots \\ 0 & 0 & 0 & \cdots & 1 \end{array}\right),\;\;\mathrm{where}\;\;a_{ii}=1-{}^{1}\;{/}_{\frac{L}{N}},$$

with *L* and *N* the length of the observation sequence and the model size, respectively. In Equation (2), instead of initializing the transition probabilities with an arbitrary initial guess and subsequently applying the Baum–Welch (BW) optimization algorithm [31], we choose to initialize the self-transitions probabilities, so they will better represent the state duration, which, in turn, will improve the model response for letters with a relatively big number of self-transitions, such as ﺍ, ب, ت, ث and ل. Then, the next state transition probability is defined in terms of the self-transition probability.

The second HMM parameter that should be initialized is the emission matrix *B*, indicating the probability of emission of a symbol (a quantization level) when the model is in a given state. Since we adopted discrete HMM to build the letter models and inspired by the work of [32], we assume that every quantization level has an equal chance of being emitted by any state; hence, $b_{ij}\in B$ are assigned an equal probability value, and consequently, the entire emission matrix is constructed according to the following equation:

(3) $$B=\left(\begin{array}{cccc} b_{11} & b_{12} & \cdots & b_{1M}\\ b_{21} & b_{22} & \cdots & b_{2M}\\ \vdots & \vdots & \ddots & \vdots \\ b_{N1} & b_{N2} & \cdots & b_{NM} \end{array}\right),\;\;\mathrm{where}\;\;b_{ij}={}^{1}\;{/}_{M},$$

where *M* is the total number of quantization levels and *N* is the length of the observation sequence. Finally, the vector of initial state probability $\pi =\{\pi_{i}\}$ that holds the probabilities of initial states is initialized as follows:

(4) $$\pi ={\left(\begin{array}{cccc} \pi_{1} & \pi_{2} & \cdots & \pi_{N} \end{array}\right)}^{T},\;\mathrm{where}\;\pi_{1}=1,\;\mathrm{and}\;\forall \pi_{i}=0,$$

which implies that the process of the training will start for every model from the first state.

### 5.3. HMM Models’ Construction

After optimization and initialization of the HMM model parameters (λ=(π,A,B)), a Baum–Welch algorithm (BW)-based approach is used to train two different kind of models [3]. The first is what we called reference models $\lambda_{R}$ built using the $f_{a}$ feature sequence, and the second is the so-called confirmation models $\lambda_{C}$ constructed using $f_{c}$ feature sequences. Basically, for each letter, in every shape, a set of training data consists of sequences of observed features $O=(o_{1},o_{2},\ldots ,o_{T})$ used to iteratively calculate a better estimation of the model parameters. The training procedure is iterated until convergence is reached or a pre-set maximum number of iteration is exceeded. In our system, the training process is regarded as converged, if the inequality (Equation (5)) is satisfied, where (ϵ=0.001) is a designated tolerance value, which indicates minimum changes in the model parameter values. Alternatively, training is considered as converged, if a maximum number of iterations (i.e., 500) is exceeded.

(5) $$\sum_{i=1}^{N}\sum_{j=1}^{N}|{\hat{a}}_{ij}-a_{ij}|\;+\sum_{j=1}^{N}\sum_{m=1}^{M}\left|{\hat{b}}_{jm}-b_{jm}\right|\;<\;\epsilon ,$$

where *N* is the model size, *M* the number of the quantization levels and $\hat{a}$ and $\hat{b}$ are the previous estimation of transition and observation probabilities, respectively. As a result of the training process, a total number of 122 reference models and 122 confirmation models are built and saved to be used in the recognition process.

### 5.4. Threshold Models’ Construction

The high variability of handwriting prevents a perfect segmentation of handwritten words. Consequently, segments with almost infinite shape variations may be passed for recognition; where the HMM approach will calculate scores indicating how well a given segment matches the different models. Due to the fact that HMM works by maximizing the segment likelihood over all models, there is no way to reject an out-of-vocabulary or a meaningless segment, which in turn increase the probability of insertion errors. As a result of weak matching between a meaningful segment and a model, a segment might be wrongly assigned to a model, causing the so-called substitution errors. Therefore, the selection of proper threshold values is very critical for the recognition system performance. If a very low value is used as a threshold, we risk accepting a large number of out-of-vocabulary segments (i.e., increase in false-positive errors), and if the threshold is set to a very high value, valid segments may be rejected (i.e., increase in false-negative errors).

Inspired by the idea of the garbage model in speech recognition [33] and a similar idea for gesture recognition [34], we propose an adaptive HMM-based solution for offline handwriting recognition called the threshold model, upon which we calculate likelihood threshold values. According to the left-to-right banded topology, each model has two types of transition probabilities, namely self-transition and forward transition. Typically, the former represents an integral pattern within the modeled letter shape, and the latter represents a shared transitional pattern.

**Figure 5:**
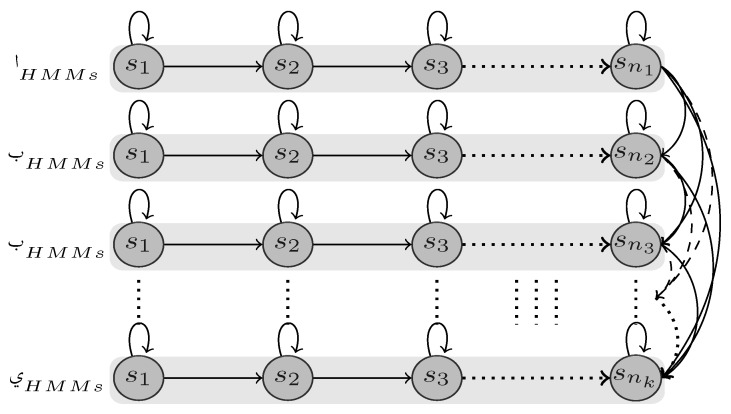
A plain structure of the HMM threshold model.

Due to this trait, we built our threshold model by copying all states of all models and ergodically connecting them all in one big model (Figure 5), so each state can be reach by all other states. In the new model, the self-transition probabilities and emission probabilities will retain the same values as in the original models, whereas the outgoing transition probabilities will be updated according to the following equation:

(6) $$a_{ij}=\frac{1-a_{ij}}{M-1},\;\;\forall j,\;i,\;\mathrm{and}\;i\neq j,$$

where $a_{ij}$ is the transition probability from state $s_{i}$ to $s_{j}$ and *M* is the sum of states number over all letters. Adopting the states along with their emission probabilities and self-transition probabilities makes them capable of representing any pattern of the modeled segments. Moreover, the ergodic connectivity allows the model to capture any random combination of sub-patterns that may result from the segmentation process.

Interestingly, however, the likelihood of a modeled segment over the threshold model will be always less than that calculated against its dedicated model, since the outgoing transition probabilities are significantly reduced according to Equation (6). Therefore, we use the likelihood calculated upon the threshold model as an adaptive threshold, i.e., a segment is assigned a model label, if and only if its likelihood upon any model is greater than that generated from the threshold model.

In general, two different types of threshold models ($\lambda_{R_{t}}$ and $\lambda_{C_{t}}$) have been constructed, the first to be used with reference models and the second with conformation models in the recognition process, which we will present in the next subsection.

### 5.5. HMM-Based Recognition

The proposed HMM-based recognition system is constructed by combining the aforementioned HMM models at the decision level. The classifier consists of two sub-classifiers, the reference-based classifier ($\lambda_{R}$), which is in turn composed of the dedicated models and the corresponding threshold model ($\lambda_{R_{t}}$), and the confirmation-based classifier ($\lambda_{C}$), which is similarly built from the dedicated conformation models and their threshold model ($\lambda_{C_{t}}$).

After the shape-based taxonomization and as an input, the classifier receives simultaneously two different sequences of observations, $O_{Fa}$ (anticlockwise features) and $O_{Fc}$ (clockwise features), that are passed to the respective classifier (reference or confirmation). Assigning a label to an observation sequence is usually regarded as an HMM evaluation problem. Instead of using the common forward algorithm to solve this problem, we adopt a more efficient and less-expensive alternative, i.e., the Viterbi algorithm [31,35], which is often used to solve the HMM decoding problem. Typically, the forward algorithm computes the probability of an observation sequence, given a model over all possible state sequences, whereas using the Viterbi algorithm, the same probability will be estimated only using the single most likely path of states within the model. Using the Viterbi algorithm, we first estimate the most likely sequence of states P, then for each model, we calculate the probability P(O,P|λ), where O and λ are an observation sequence and an HMM model, respectively. For reference and confirmation models, the recognition problem is regarded as a scoring problem, where dedicated letter models are competing, and the label with the maximum probability will be picked as an intermediate recognition result. Before reaching a definitive recognition decision, combinations of intermediate results are performed on two levels. Firstly, an intermediate result is computed from the dedicated models and the corresponding threshold model. Then, the final result is estimated by combining the intermediate results of anticlockwise- and clockwise-based models. Strictly speaking, a segmented handwritten stroke can be successfully classified, given the following: Firstly, we use the Viterbi algorithm to compute the following intermediate logarithmic probabilities (computed values can become very small; hence, logarithmic calculation is used to avoid arithmetic underflow errors).

- $L_{R}=\log\left(\max_{1\leq i\leq N_{T}}\left[P\left(O_{Fa},P_{R_{i}}|\lambda_{R_{i}}\right)\right]\right),\;\mathrm{and}\;$$L_{Rt}=\log\left(P(O_{Fa},P_{Rt}|\lambda_{Rt})\right)\;$. $L_{R}$ is the highest probability over all reference models, where $\lambda_{R_{i}}$ and $P_{R_{i}}$ are the involved model and its associated most likely path. $L_{Rt}$ is the probability of the same observation sequence computed against the reference threshold mode and $N_{T}$ is the number of models of considered taxonomy.
- $L_{C}=\log\left(\max_{1\leq j\leq N_{T}}\left[P\left(O_{Fc},P_{C_{j}}|\lambda_{C_{j}}\right)\right]\right),\;\mathrm{and}\;$$L_{Ct}=\log\left(P(O_{Fc},P_{Ct}|\lambda_{Ct})\right)$. Similarly $L_{C}$ and $L_{Ct}$ are estimated as above except that confirmation models and confirmation threshold model are used instead of their reference counterparts.

Secondly, and because of the fact that the confidence measure of the results typically makes the recognition systems more useful in real-time applications [33,36], the definitive recognition results are returned along with confidence values.

Given the above estimated probabilities for a handwritten stroke, four different outcomes are expected depending on the following inequalities:

- if $L_{R}>L_{Rt}$ and $L_{C}>L_{Ct}$, where both $L_{R},L_{C}$ refer to the same label (i.e letter) in reference as well as in confirmation models, the label will be assigned to the stroke assuming complete confidence.
- if $L_{R}>L_{Rt}$ and $L_{C}>L_{Ct}$, yet $L_{R},L_{C}$ point out to different labels, then the label of higher probability is assigned to the stroke, and a substitution error is reported (i.e., $\;S_{error}=S_{error}+1$).
- if $L_{R}\leq L_{Rt}$ or $L_{C}\leq L_{Ct}$, then the label corresponding to the one with probability higher than that of its own threshold model, will be picked as a recognition result, and an insertion error will be reported (i.e., $\;I_{error}=I_{error}+1$).
- if $L_{R}\leq L_{Rt}$ and $L_{C}\leq L_{Ct}$, then the stroke will be rejected and a deletion error will be reported (i.e., $\;D_{error}=D_{error}+1$).

As we previously mentioned, the inputs of the recognition system are observation sequences representing the various segmented strokes that constitute a given handwritten word; consequently, the individual recognition outputs will be combined altogether to form the recognized word. The overall recognition confidence will be calculated as follows:

(7) $$conf=1-\frac{0.5S_{error}+0.5I_{error}+D_{error}}{N},$$

where $S_{error},\;I_{error}$ are weighted at 0.5 and N is total number of segmented strokes of the word. Typically, the recognition results of the proposed system are a sequence of UNICODE letters with an overall confidence value; however, if a segment is regarded as out-of-vocabulary, an # will be returned instead.

## 6. Linear Chain CRF-Based Recognition of Arabic Handwriting

As mentioned in the Introduction, to predict a sequence of letter class labels y for a given vector of feature observations x, most of the previous related works focused on the generatively-trained HMM. To reduce the model complexity, HMM is based on the assumption of conditional independence among input data, which might reduce the model accuracy [6]. CRF and its extension HCRF were both introduced to address this shortcoming, where dependencies are assumed among labels without presuming any kind of dependency between observation sequences [13,37,38]. In our approach, we assume that letter class labels y are fully observed, where each $y_{i}\in y$ represents a class label of a basic shape in which a letter may appear. By using the anticlockwise features $f_{a}$ and the clockwise features $f_{c}$, two different exponential linear-chain CRF models are respectively created for each node, where every letter’s “basic” shape under the concerned node has a corresponding state within the model. Furthermore, the proposed models are typically built using two different types of feature functions, the transition feature functions $t(y_{i-1},y_{i},x,i)$ and the emission feature functions $s(y_{i},x,i)$. These functions take as input an entire sequence of observations x, the current position within the sequence *i*, the current class label $y_{i}$ and the previous class label $y_{i-1}$ and output a real-valued number.

Transition functions are typically dedicated to estimating the dependency of neighboring class labels given the value of the current position in x. The emission functions are employed to estimate real values to represent the possibility that the current label emits the current value in x. In our approach, to calculate the sequence overall likelihood, firstly, the transition and emission functions are assigned the weights γ and μ, respectively, which are learned from the training data. Then, they are combined together to form a potential function as follows:

(8) $$F_{\theta }\left(y_{i-1},y_{i},x,i\right)=\sum_{f}\gamma_{f}t_{f}\left(y_{i-1},y_{i},x,i\right)+\sum_{g}\mu_{g}s_{g}\left(y_{i},x,i\right),$$

where $\theta =(\gamma_{1},\gamma_{2},\ldots ,\gamma_{N_{f}};\mu_{1},\mu_{2},\ldots ,\mu_{N_{g}})$, $\gamma_{i}\in \gamma$, $\mu_{i}\in \mu$ and $N_{f}$ and $N_{g}$ are the total number of feature functions and emission functions, respectively. Finally, to convert the outputs of $F_{\theta }$ into proper probabilities, $F_{\theta }$ is summed over all $x_{i}\in x$, and the result is exponentiated and normalized as follows:

(9) $$p_{\theta }\left(y|x\right)=\frac{\exp\left(\sum_{i=1}^{n}F_{\theta }\left(y_{i-1},y_{i},x,i\right)\right)}{Z_{\theta }\left(x\right)},$$

where the normalization factor $Z_{\theta }\left(x\right)$ is given by:

(10) $$Z_{\theta }\left(x\right)=\sum_{y}\exp\left(\sum_{i=1}^{n}F_{\theta }\left(y_{i-1},y_{i},x,i\right)\right).$$

Additionally, to investigate the effect of the dependency range on the performance of the proposed system and in addition to the θ parameter, the potential function $F_{\theta }$ is also parametrized by an ω window size parameter. The additional parameter is defined to capture the number of previous and subsequent observations used when predicting a class label at the current position *i*, (e.g., for a window size of ω, the observation from i−ω to i+ω will be used to calculate the outputs of $F_{\theta }$). To optimize the ω size for each node, we trained and experimented with seven CRF models corresponding to seven different window sizes (ω=0,ω=1,…,ω=6).

### 6.1. CRF Parameter Learning

To learn the CRF feature functions weights θ, we applied the popular gradient ascent algorithm on fully-labeled training sequences $D={\left\{\left(x^{t},y^{t}\right)\right\}}_{t=1}^{T}$, where $x^{t}$ is a sequence of observed features, $y^{t}$ is the corresponding sequence of labels and *T* is the total number of training samples. The learning process starts by randomly initializing θ, then for each training sample and for each potential function, the gradient of the log probability with respect to θ is calculated as follows:

(11) $$\frac{\partial L(\theta )}{\partial \theta }=\sum_{t=1}^{T}\left(\sum_{i=1}^{n}\frac{\partial F_{\theta }\left(y_{i-1}^{t},y_{i}^{t},x^{t},i\right)}{\partial \theta }\;-\sum_{x}p_{\theta }\left(y|x^{t}\right)\sum_{i=1}^{n}\frac{\partial F_{\theta }\left(y_{i-1},y_{i},x^{t},i\right)}{\partial \theta }\right),$$

where $L\left(\theta \right)=\sum_{t=1}^{T}\log\;p_{\theta }\left(y^{t}|x^{t}\right)$, and the first term in the gradient is the contribution of $F_{\theta }$ under the true label, whereas the second term is the expected contribution of $F_{\theta }$ under the current model. The well-known L-BFGSalgorithm [38,39] is used for the gradient calculation, and the convergence is assumed to be reached within 300 iterations.

### 6.2. Class Label Prediction

According to the proposed CRF approach, a sequence of inputs is recognized, first by labeling each element in the sequence through calculating the corresponding optimal Viterbi path under the considered CRF model. Then, the most frequently-occurring class label along the sequence of predicted labels is chosen as an intermediate prediction. As discussed at the beginning of this section, the proposed CRF classifier consists of two sub-classifiers: the first is dedicated to predict labels for $f_{a}$ features; and the other is to predict labels for $f_{c}$ features. Therefore, we proposed that the ultimate recognition decision be jointly decided by the respective results of the two sub-classifiers. Moreover, and since the number of the labels to be predicted is relatively high, simply picking the most frequent label as the prediction is not enough for a reliable recognition. Thus, we propose a threshold of minimum-occurrence ($\varepsilon_{\bar{L}}$) of a label $\bar{L}$, to be chosen as the unique global label of the entire sequence. Empirically, the best recognition result is achieved at an average of $\varepsilon_{\bar{L}}\approx 40\%$; hence, $\varepsilon_{\bar{L}}=40\%$ is chosen as the minimum-occurrence threshold.

Given a class label ${\bar{L}}_{a}$ that occurs $k_{a}$ times along a sequence of to be predicted $f_{a}$ and a class label ${\bar{L}}_{c}$ that occurs $k_{c}$ times a long a sequence of to be predicted $f_{c}$, the proposed CRF system recognizes a segment by combining the intermediate results of the two subsystems as follows: Similar to the HMM approach, recognition of a handwritten word is considered equivalent to the process of recognizing each of its segments, and the attached values indicating the error possibilities are combined to form a word-based confidence score, as in Equation (7).

## 7. HCRF for Arabic Handwriting Recognition

It is commonly agreed that models with a hidden-state structure usually outperform fully-observed ones, since they are more capable of capturing the relevant hidden structure in the given domain [31,40]. To investigate the performance of the probabilistic discriminative models with hidden states for offline Arabic handwriting recognition, we introduce the relatively recently-proposed HCRF [41]. HCRF is simply an extension of the fully-observed CRF model, where the HCRF model is equipped with an intermediate set of hidden variables $h=\{h_{1},h_{2},\ldots ,h_{n}\}$ (between the observations and labels), globally conditioned on the observation vector x. The hidden variables or the hidden states in our case are devoted to capturing assumed hidden patterns of observation values within the observation sequences, which may represent specific shape peculiarities along the segment.

To optimize the number of hidden states for each HCRF model, an approach similar to that employed for the HMM is adopted, where the number of hidden states is chosen to be proportional to the letter shape complexity. Accordingly, the numbers of the optimized hidden states were found to be 5, 8, 10, and 10 for Node 1 to 4, respectively. Analogous to the formulation of CRF, HCRF estimates the conditional probability of a class label y given a sequence of observations x, as follows:

(12) $$p_{\theta }\left(y,h|x\right)=\frac{\sum_{h}\exp\left(\sum_{i=1}^{n}F_{\theta }\left(y_{i-1},y_{i},h,x,i\right)\right)}{\sum_{y,h}\exp\left(\sum_{i=1}^{n}F_{\theta }\left(y_{i-1},y_{i},h,x,i\right)\right)},$$

where the denominator is a normalization factor similar to $Z_{\theta }\left(x\right)$ in Equation 9 and the potential function $F_{\theta }\left(y_{i-1},y_{i},h,x,i\right)$ computes the similarity between a class label, a sequence of observations and a configuration of hidden states. As previously stated, HCRF is a slightly modified version of CRF; hence, parameter learning performed similar to that of CRF, where the optimal set of weights θ for each node is calculated upon the set of training samples $D={\left\{\left(x^{t},y^{t}\right)\right\}}_{t=1}^{T}$ using the L-BFGS algorithm [39]. Furthermore, convergence is also assumed to be reached within 300 iterations. As a result of the training, seven different HCRF models are built for each node, each with different window sizes (i.e., ω=0,ω=1,…,ω=6). Finally, label prediction, overall recognition results and the attached confidence scores are all computed just like in the CRF approach.

## 8. Experimental Results

Since handwritten words samples of the IESK-arDBdatabase [2] are all annotated with segmentation information, we used this database to train the HMM, CRF and HCRF classifiers. A total of 800 handwritten words containing about 3500 letters were used to build letter models and taxonomy models (in the case of CRF and HCRF). In the evaluation experiments, we used not only 400 words images containing more than 1700 letters from the IESK-arDB database, but also 200 (manually segmentation and ground-truthed) word images with about 1000 letters from the IFN-ENIT database [42]. The proposed systems were implemented in MATLAB and C++, where the MATLAB HMM toolbox and the HCRF library (include CRF) were used [41]. All experiments were performed on a Windows 7 professional and MATLAB R2013a installed on an Intel(R) Xenon(R) CPU server machine with 2.67 GHz and 64.0 GB of memory. The recognition performance of the proposed systems was sufficiently evaluated with respect to both letters and words.

### 8.1. Evaluation of HMM Recognition Performance

According to our HMM recognition approach, a segment image was first assigned to one of four shape-based taxonomies. Then, features were extracted and tested against the corresponding set of HMM models and the respective threshold model. As a result, a letter might have been either recognized with a complete confidence value, recognized with a 50% possibility of a substitution error ($S_{error}$), recognized with a 50% possibility of insertion error ($I_{error}$) or completely rejected, and hence, a deletion error would have been reported.

Because of their relative distinct shapes and the absence of additional dots and diacritics, letters such as (ﺍ د ر و ه) achieved high recognition rates, compared to letters consisting of multiple strokes (e.g., ت ث ذ ز).

**Figure 6:**
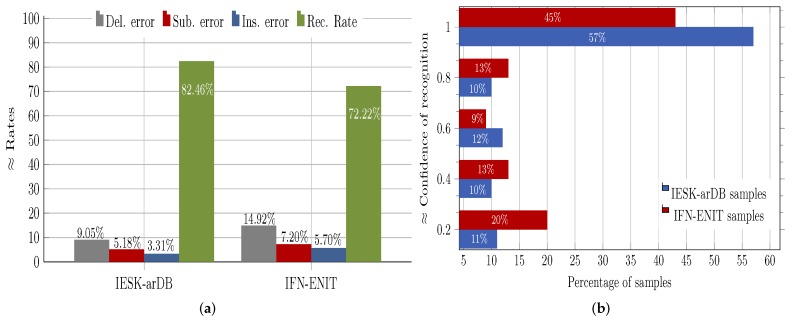
Recognition rates obtained on letters and words: (**a**) recognition achieved on letter samples from two different databases along with observed classification errors; (**b**) percentage of recognized words with the respective recognition reliability values.

This was mainly caused by shifting and/or fusion of dots and diacritics, which significantly increase shape similarity and weaken the features’ discriminative power. Further, it was observed that the letter position (i.e., the written form) inside a word affected the recognition performance, where, for example, letters in isolation form achieved the highest recognition rate (i.e., 85.39%), while letters written in the middle form achieved the lowest rate (i.e., 79%). Figure 6a presents the overall letter-based performance of the proposed HMM recognition approach on the IESK-arDB and IFN-ENIT evolution sets and further explains the amount and the type of errors occurring during the classification process. Basically, three different types of errors have been observed. The first was the deletion error, which occurred when a letter was not recognized, because its maximum likelihood was less than that of the threshold model. The second type of errors was the so-called substitution error, which was generated when a letter was confused with another one. The last error was the insertion error, which was the result of recognizing a non-letter segment (resulting from inaccurate segmentation) as a letter. On the IESK-arDB letter evaluation set (as depicted in Figure 6a, an average recognition rate of 82.28% was reached, whereas 9.24%, 5.18% and 3.30% were reported as deletion, substitution and insertion error rates, respectively. In comparison to the results achieved using IESK-arDB, the average results obtained on the IFN-ENIT evaluation set were relatively weaker (i.e., 72.22%, 14.92%, 7.20% and 5.70%, for recognition rates, deletion errors, substitution errors and insertion errors, respectively), which can be attributed to the fact that letter models were built using only samples from the IESK-arDB.

Instead of rejecting or recognizing a handwritten word as whole, in the proposed approach, the word recognition problem was reformulated into tractable sub-problems of rejection or recognition of the letters constituting the considered word. Furthermore, a confidence value (conf∈[0,…,1]) was attached to the recognition results to describe how reliable the result was, where conf=0 meant a non-recognizable word, while conf=1 implied a complete confidence in the recognition result. Besides being helpful in the assessment of the reliability of the recognition results, attached confidence values can also be used to initiate post-processing procedures (e.g., as spelling and grammar correction), which may further improve the obtained recognition results.

Table 1 gives some examples of handwritten word images along with the Unicode letters and the corresponding recognition confidence values. Figure 6b shows the achieved results when the proposed HMM approach was tested on the evaluation sets of the IESK-arDB and IFN-ENIT databases. For simplicity purposes, results were projected into an axis of five different pins of confidence values, and each result was sorted by rounding its confidence value to the nearest pin value. On the words’ level, recognition associated with confidence values higher than 0.5 was 79% and 67% for samples of the IESK-arDB and IFN-ENIT databases, respectively. On the other hand, a low confidence of less than 0.3 was reported for 11% of samples of the IESK-arDB samples and for 20% of the IFN-ENIT samples. We believe that such results can be improved by both further optimizing the HMM model parameters and integrating a spelling checker as a post-processing stage.

Finally, it is also important to mention that the relatively poor performance on IFN-ENIT samples is related to several facts, such as: (i) the models were only constructed from the samples drawn from the IESK-arDB database; (ii) IFN-ENIT samples consist very often of multiple words with excessive elongation, which complicated the segmentation process; and (iii) the variability of IFN/ENIT was higher than that of IESK-arDB, as more writers were involved, and diacritics such as SHADA, which is not popular in handwriting, were added to the letter main body.

### 8.2. Performance Evaluation of CRF and HCRF

Instead of building a model for each class as in HMM, CRF and HCRF work by building a single model for all to be predicted classes, where each class was represented by a single state within the model. Accordingly and in order to keep the number of states manageable, we created CRF and HCRF models separately for each node. Moreover, models ’training and testing were performed using the same datasets used for the HMM approach.

**Figure 7:**
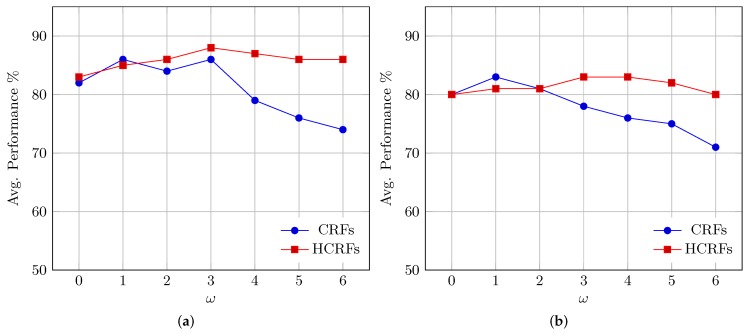
CRF and HCRF performance with different window sizes (ω): (**a**) performance on Tax.2; (**b**) performance on Tax.3.

Experiments were conducted to fulfil two purposes, firstly to optimize the parameters of the respective recognition system and secondly to test the system efficiency. To optimize the window-size parameter for each taxonomy, we trained seven CRF and seven HCRF models for each taxonomy, where every model was trained using different window sizes (ω=0,ω=1,…,ω=6). Generally, 28 CRF and 28 HCRF models were built, and by individually evaluating the performance of each model, only the best performing CRF (one) and the best performing HCRF models were selected for each taxonomy. Figure 7 compares the performance of CRF and HCRF and shows the effect of modeling the dependency range by using different window sizes (ω) on Tax.2 and Tax.3 as examples. The experimental results indicate that in the case of CRF, only incorporating the direct neighbors (i.e., ω=1) will positively affect performance, whereas completely ignoring neighboring elements in the computation (i.e., ω=0) or considering faraway elements (i.e., ω>1) drastically decreased the system performance. When assessing the performance of the CRF models using the letter-based IESK-arDB test set, the best recognition rates were achieved through classifiers built with ω=1; hence, only models built with ω=1 were selected as CRF representatives for the rest of the experiments. 

As for HCRF, by using the same test set, the performance improved as ω increased, reaching its peak over all taxonomies at ω=3, then beginning to decay beyond ω=4. Such a tendency implies that incorporating dependencies positively influences performance, especially when the hidden pattern is also reasonably considered.

Table 2 summarizes the average letter-based results achieved using the best-performing CRF (i.e., with ω=1) and the best-performing HCRF (i.e., with ω=3). In general, the achieved results confirm the fact that recognition performance is inversely proportional to the number of the classes. Therefore, the lowest recognition rates were reported when testing CRF and HCRF of node Tax.3, with average rates of 82.10% and 83.64%, respectively. Such a modest performance can be explained by the relatively large number and the complex shape characteristics of letters falling under Tax.3. Contrary to results achieved on Tax.3, CRF and HCRF reached their best results of 84.88% and 87.00%, respectively, on the test samples of node Tax.2, which had the fewest number of class labels.

**Figure 8:**
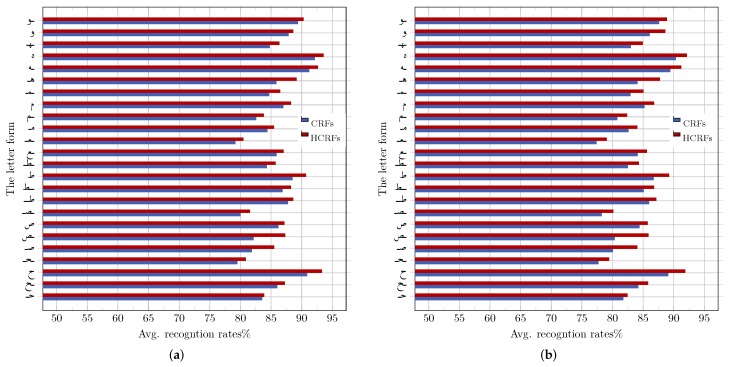
CRF and HCRF performance on letter shapes under node Tax.2: (**a**) performance on samples drawn from the IESK-arDB database; and (**b**) performance on samples drawn from the IFN-ENIT database.

For more insight and as an example, Figure 8 details the average recognition rates achieved on letters falling under node Tax.2 (i.e., letters of one stroke with a loop), for both the IESK-arDB and IFN-ENIT databases. As expected, letter forms with distinctive shapes such as isolated “ه” and “ح” were recognized efficiently, since they are less likely to be confused with other class labels and also very often perfectly segmented (notice that “ح” is classified under Tax.2, since it is often handwritten with an upper loop). Figure 8a, shows that the CRF and HCRF approaches reached their best recognition results (i.e., 92.13% and 93.58%, respectively) on the simple shape of letter “ه”.

**Figure 9:**
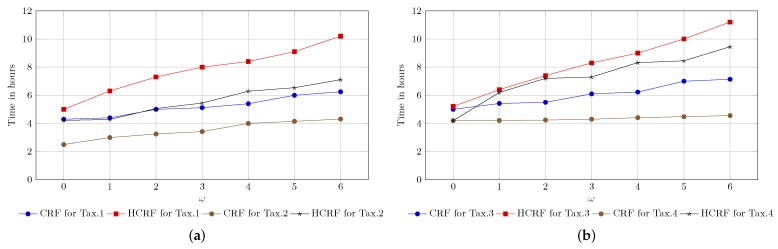
CRF and HCRF training cost in terms of time: (**a**) training cost of CRF and HCRF for Tax.1 and Tax.2; (**b**) training cost of CRF and HCRF for Tax.3 and Tax.4.

On the other hand, CRF and HCRF demonstrated the weakest performance on the middle form of letter “ع”, where 79.51% and 80.48% are respectively registered for CRF and HCRF on samples of IESK-arDB (see Figure 8a) and 77.41% and 79.05% for samples of the IFN-ENIT database (Figure 8b). Such a tendency can be attributed to the fact that “ ﻌ ” is usually written with a middle loop, which makes it vulnerable to being confused with other letters forms, such as “ ﻤ ”, “ ﺼ ” and “ ﺤ ”. It is also important to point out that, on average, performance varies according to the considered letter form, where the highest recognition rates of 86.42% and 87.93% were reached on the isolated form for CRF and HCRF, respectively. The lowest rates of 80.79% and 82.48% were obtained on the middle form for both classifiers, respectively. This is due to the fact that letters in the isolated form are typically separated by a white-space from the neighboring letters. This led to a perfect segmentation, hence increasing the discrimination of the extracted features. Figure 9 summarizes the obtained results according to the four different handwritten forms, where the HMM, CRF and HCRF classifiers all reach their best performance on letters in the isolated form.

**Figure 10:**
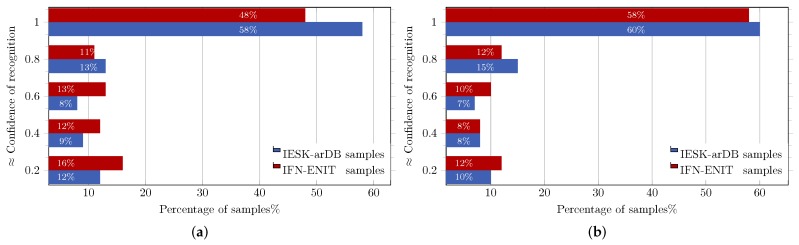
CRF and HCRF word-based performance on evaluation sets of the IESK-arDB and IFN-ENIT databases: (**a**) CRF performance; (**b**) HCRF performance.

Similar to the HMM approach, word recognition using CRF and HCRF was also performed as the result of recognizing each stroke in the considered word. To indicate how reliable the recognition result was, an overall confidence values was calculated and attached to the recognition results. Figure 10a,b, details the results achieved using CRF and HCRF, respectively. For simplicity, the obtained results are rounded and projected into five different levels of confidence. When tested, the CRF system recognized 79% of word images of IESK-arDB and 72% of word images of FIN-ENIT, with confidence values higher than 0.5. On the other hand, 82% and 80% of samples from IESK-arDB and IFN-ENIT were recognized respectively with confidence values higher than 0.5 using the HCRF system. Results with confidence values less than 0.5 were considered to be a low recognition rate (which registered 21% of IESK-arDB samples and 28% of IFN-ENIT samples using the CRF approach and, similarly, on 18% and 20% using HCRF). Considering the obtained recognition results, we can conclude that the HCRF recognition approach clearly outperforms the CRF one, specially on IFN-ENIT samples, as the HCRF hidden layer allows the model to be potentially adapted to any unseen handwriting pattern, and hence, a performance boost of about 8% was observed.

However, the HCRF was very expensive in terms of training costs. Figure 9b summarizes the cost in terms of time for the two approaches on all taxonomies and further indicates that the cost is proportional to the window size ω, the size of the considered node (i.e., the number of different class labels) and to the number of hidden states in the case of HCRF. As an example, the process of training HCRF model for Tax.3 with window size ω=5 and 10 hidden states, required 10 h on average, while the same process needed about 9 h for Tax.1.

### 8.3. HMM vs. CRF vs. HCRF

In order to ensure a fair comparison between the HMM, CRF and HCRF approaches, all three approaches were trained and tested using the same training and evaluation datasets. Further, the recognition results of the three approaches were presented on the letter, as well as on the word level, where the same metric was used in the evaluation process. In this subsection, we compare the performance of each approach to the rest, highlighting the strengths and weaknesses, and give recommendations for a possible future application of each approach.

**Figure 11:**
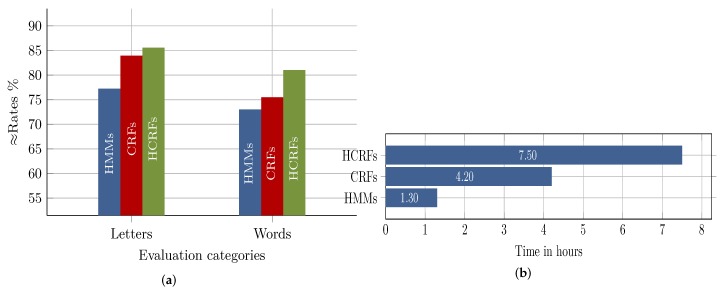
Overall recognition performance and training costs in terms of time: (**a**) the average performance of the three approaches for the recognition of segmented letters and for recognized words with confidence values greater than 0.5; (**b**) the average time needed to model letters of one node.

The first part of Figure 11a summarizes the average recognition rates of the three approaches on letters. Due to the discriminative-based training and the modeling of the hidden pattern through the hidden sates, HCRF achieved the best performance rates of 85.60%, followed by the discriminatively-trained CRF, which achieved 84.0%, and the generative HMM, with a performance of 77.30%. The obtained recognition rates, firstly, confirm the efficiency of the discriminative models compared to the generative HMM and, secondly, indicate the slight improvement in performance when the hidden states layer was introduced to CRF (i.e., HCRF). Compared to the results achieved by several approaches in [43], who participated in the competition of the International Conference on Document Analysis and Recognition (ICDAR 2009), our results are quite satisfactory and encouraging for two reasons: (i) the competing recognition systems considered only the isolated form of letters; hence, only 30 class labels (at maximum) were dealt with, compared to processing 122 class labels in our approaches, where each letter form was modeled; (ii) our letter models were built using letter shapes segmented directly from handwritten words rather than from isolated well-written letters, which makes our approaches more applicable in realistic situations.

The second part of Figure 11a illustrates the percentage of handwritten words that were partially or completely recognized with confidence values higher than 0.5. The three approaches show similar behavior as in the letter recognition case. The HCRF classifier attained the best performance of 81%, while CRF and HMM achieved a performance of 75.5% and 73%, respectively. Even though the word-based recognition results were still unsatisfactory, especially the results with confidence values less than 0.8, we believe the state-of-the-art spell correction solutions such as MS spell checker, Google spell checker or Hunspell for Arabic can be used to improve performance significantly [44].

On average, CRF and HCRF showed a strong performance compared to HMM; however, both were very expensive in terms of time and computation costs. Figure 11b shows the average time needed for each approach to model a set of letters under one node. It is obvious that the HMM approach was significantly time efficient compared to CRF and HCRF. Furthermore, the high time costs of HCRF compared to CRF should also be considered and justified by important performance improvements. We believe that training costs are an important factor that should be considered especially in the case of the CRF and HCRF approaches. This is because of the fact that CRF and HCRF, basically, model a classification problem by creating one model in which an individual class or label is represented through a state. Consequently, any update such as adding or removing a class or label would require re-training the model. This is especially true for holistic-based OCR systems, where the OCR problem is constrained to a limited and fixed lexicon.

Finally, we should emphasize the following points: (i) Despite the fact that in our experiments, both the HCRF and CRF approaches outperformed the HMM, we expect, however, in application-specific small lexicon-based solutions, such as in banking and postal sectors, that the gain in performance might not justify the choice of the expensive HCRF or CRF. Thus, in such cases, we recommend to begin by investigating the performance of an HMM solution. (ii) For unconstrained solutions with large or unlimited lexicons, and rather than directly deciding on an HCRF-based solution, we strongly recommend firstly assessing the performance of the reasonably performing and less expensive CRF-based approach.

## 9. Summary and Conclusions

The paper’s main objective is to compare the performance of generative and discriminative probabilistic classification models on offline Arabic handwriting. Instead of using the well-known sliding window-based features, the paper begins by introducing new shape description features, which is cost-effective, yet capable of capturing discriminative shape characteristics. Moreover, due to the fact that performance is inversely proportional to the number of classes, letters are shape-based grouped into four categories, which significantly reduced the class-space. As the generative recognition approach, we trained an HMM equipped with adaptive threshold models. The threshold models were built to allow HMM to reasonably reject out-of-vocabulary segments and meaningless patterns. Alternatively, we introduced the relatively recently proposed discriminative classifiers, namely the CRF and its extension, HCRF. For a fair comparison, all classifiers (i.e., HMM, CRF, and HCRF) were trained and tested on the same datasets drawn from two different databases. Besides the achieved results on both letter, as well as on word levels, deletion, substitution and insertion errors were reported and discussed. Finally, the performance comparisons and discussion of the pros and cons of each approach were also given.

Offline handwriting recognition in general and Arabic handwriting in particular is still an active field of research, where many challenges still wait for novel ideas and much continuous effort is to be overcome. In this context, we believe that the label-space can be further reduced, since several letters sharing the same basic shape and are only distinguishable through diacritics. On the feature extraction and classification levels, experimenting with different types of features, a better optimization of the involved models’ parameters and integrating spell correction algorithms are all possible future enhancements.

Recently, deep learning has emerged as a powerful learning technique, with outstanding success in many complex pattern recognition fields. The main obstacle that hinders efficient application of deep learning in the field of offline handwriting recognition is the lack of comprehensive and adequate databases needed for training. In a previous work [45], we proposed a handwriting synthesis approach to overcome this problem. Hence and as future work, we are excited to experiment with deep learning methods such as the Multi-Dimensional Long Short-Term Memory Recurrent Neural Networks (MDLSTM-RNNs) [46,47], which recently became the state-of-the-art approach for sequence labeling-based problems like handwriting recognition. 

## Figures and Tables

**Table 1:** Examples of the inputs and the results of the HMM-based recognition approach. Notice in the third row the third segment (from the right) is rejected; hence it is replaced with #. Furthermore, the first segment (from the right) in the fourth row is recognized as “ر” with a 50% possibility of substitution error.

Inputs	Results	
	Recognized Letter	Confidence Value (Conf.)
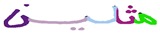	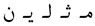	1 − (0.5 × 0 + 0.5 × 0 + 0)/5 = 1.00
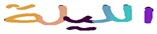	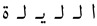	1 − (0.5 × 0 + 0.5 × 0 + 0)/6 = 1.00
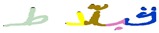	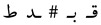	1 − (0.5 × 1 + 0.5 × 1 + 1)/5 = 0.60
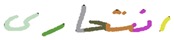	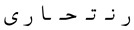	1 − (0.5 × 1 + 0.5 × 0 + 0)/7 = 0.93

**Table 2:** Letter-based performance of CRF and HCRs across the IESK-arDB and IFN-ENIT databases for the four-letter taxonomies.

Dataset	CRF’ Rec. Rates %				HCRF’ Rec. Rates %			
	Tax.1	Tax.2	Tax.3	Tax.4	Tax.1	Tax.2	Tax.3	Tax.4
IESK-arDB	85.83	85.77	83.37	85.24	86.87	87.45	84.73	86.65
IFN-ENIT	83.51	83.99	80.82	82.88	84.93	86.54	82.54	84.76
Avg.%	83.95				85.56			
